# Structural and functional alterations in the brain during working memory in medication-naïve patients at clinical high-risk for psychosis

**DOI:** 10.1371/journal.pone.0196289

**Published:** 2018-05-09

**Authors:** Jens Gisselgård, Alexander V. Lebedev, Kathinka Dæhli Kurz, Inge Joa, Jan Olav Johannessen, Kolbjørn Brønnick

**Affiliations:** 1 Stavanger University Hospital, TIPS- Regional Centre for Clinical Research in Psychosis, Stavanger, Norway; 2 Department of clinical neuroscience, Karolinska Institutet, Stockholm, Sweden; 3 Aging Research Center, Karolinska Institutet & Stockholm University, Stockholm, Sweden; 4 Stavanger University Hospital, Department of Radiology, Radiology Research Group, Stavanger, Norway; 5 Department of Electrical and Computer Engineering, University of Stavanger, Stavanger, Norway; 6 Network for medical sciences, University of Stavanger, Stavanger, Norway; Maastricht University, NETHERLANDS

## Abstract

Several previous studies suggest that clinical high risk for psychosis (CHR) is associated with prefrontal functional abnormalities and more widespread reduced grey matter in prefrontal, temporal and parietal areas. We investigated neural correlates to CHR in medication-naïve patients. 41 CHR patients and 37 healthy controls were examined with 1.5 Tesla MRI, yielding functional scans while performing an N-back task and structural T1-weighted brain images. Functional and structural data underwent automated preprocessing steps in SPM and Freesurfer, correspondingly. The groups were compared employing mass-univariate strategy within the generalized linear modelling framework. CHR demonstrated reduced suppression of the medial temporal lobe (MTL) regions during n-back task. We also found that, consistent with previous findings, CHR subjects demonstrated thinning in prefrontal, cingulate, insular and inferior temporal areas, as well as reduced hippocampal volumes. The present findings add to the growing evidence of specific structural and functional abnormalities in the brain as potential neuroimaging markers of psychosis vulnerability.

## Introduction

Most patients who develop a psychotic disorder usually experience a relatively long period prior to the first psychotic event with unspecific- and more specific symptoms. At the onset of psychosis, many already have experienced a loss of cognitive- and psychosocial functioning. For example, working memory (WM) is found to be reliably impaired in participants with schizophrenia [1, 2]. This finding has also been observed in individuals with clinical high risk for psychosis (CHR) and there is also evidence for a correlation between degree of WM impairment and risk of transition to psychosis [1]. In accordance with these findings, two recent meta-analyses showed that high-risk subjects already are cognitively impaired relative to matched controls [1, 3]. However, in the current literature there is considerable variation of the results in published studies regarding the risk of developing psychosis in different CHR groups. This may partly be explained by variations in the underlying psychiatric diagnoses and psychopathology, heterogeneity in inclusion criteria and the clinical characteristics of the study populations, the use of different paradigms and designs in functional imaging, and the use of different forms of image acquisition and image analysis [4]. In individual cases, no reliable neurocognitive markers have adequate sensitivity and specificity to classify the risk for transition to psychosis in CHR [5, 6]. Across specific domains, such as executive function, verbal fluency, attention, visual and verbal memory, and working memory, effect sizes in the studies published are typically small to medium [7]. Available research confirms that CHR is characterized by widespread mild cognitive deficits at a level that is intermediate between healthy individuals and those diagnosed with schizophrenia [8].

Functional magnetic resonance imaging (fMRI) has been used to investigate patterns of brain activity as a potential neurobiological marker in ‘ultra high-risk’ or ‘clinical high-risk’ (UHR/CHR) subjects. There is only a limited number of studies of WM in UHR/CHR [9–19]. Results of studies exploring abnormalities in prefrontal cortex (PFC) activity during WM tasks in patients with schizophrenia are mixed [20], with a majority of studies reporting task related decrease in activity or reduction in connectivity in or between regions thought to reflect a WM-network [3, 9, 14, 15, 17], while a few report increase of such activity in specific regions [18]. A common interpretation of increase of activity in patients with schizophrenia is that it represents a general compensatory mechanism of the brain in response to challenging tasks [21] or that it might depend on task performance [22] or reflect working memory load [23].

The majority of fMRI studies of UHR/CHR subjects show reduced activity in multiple frontal and parietal regions while performing these tasks. A meta-analysis of 10 fMRI studies by Fusar-Poli [10] indicated that reduced functional activation in prefrontal regions may represent a neurophysiological correlate of increased vulnerability to psychosis. A quantitative analysis by Dutt and colleagues using activation likelihood estimation in 5 studies of the N-back WM task, showed reduced activity in UHR/CHR in several prefrontal areas, the inferior parietal lobule, as well as in the superior temporal gyrus [9].

Most [24, 25], but not all [26] structural imaging studies of UHR/CHR report reductions in grey matter volume and cortical thickness in a number of regions, including prefrontal, medial temporal, and lateral temporal areas, also presenting a possibility to use information derived from the volumetric MRI data to solve diagnostic classification and prediction problems at the individual level [27–30]. The mechanisms leading to cortical volume and thickness reductions in UHR/CHR subjects are complex and most likely multifactorial. Thus, impaired hormonal responses [31] and abnormal neuro-inflammatory [32, 33] mechanisms may trigger pubertal neurodevelopmental disturbances in CHR [34] and thus lead to the afore-mentioned structural changes.

To summarize, previous studies suggest that UHR/CHR is associated with functional alterations in prefrontal, parietal, medial temporal and striatal regions during WM task performance. Structural and functional abnormalities appear to be localized within the frontoparietal circuits. A potential confounder in some of these previous studies is the effect of medication. Both cognitive and MRI markers are sensitive to the effects of antipsychotic medication [35, 36] which may add to the heterogeneity in the study populations and lead to conflicting findings. Finding neuroimaging indicators of psychosis vulnerability remains a challenge. Although significant differences have been seen compared with healthy samples, the differences are in general smaller than those observed in patients with early psychosis [37]. In the present study, we investigate neural correlates to CHR in a medication-naïve group compared to matched controls. We hypothesized that functional and structural alterations would be detectable in a medication-naïve group compared to matched controls and that these alterations would be similar to what have been found in previous studies with medicated patients.

## Materials and methods

### Participants

The sample consisted of a total of 78 participants, 41 CHR patients and 37 healthy controls. The recruitment process of CHR individuals in this study is based on the knowledge from the Early Detection and Intervention in Psychosis (TIPS 1 and 2) studies in Norway [38, 39]. TIPS have been successful in applying information campaigns and detection teams and have been instrumental in bringing young person’s presenting with first-episode psychotic symptoms into treatment and reducing the duration of untreated psychotic symptoms. All CHR patients were actively help-seeking and recruited through active outreach and as response to ongoing information campaigns focusing on CHR symptoms, aimed at both the general population and personnel in medical/health care services.

The healthy controls were recruited through friends and family of the research staff, as well as friends and family of the CHR-patients. Both patients and controls received a monetary compensation for participating in the fMRI session.

#### Inclusion and exclusion criteria

1) The patient is listed in the national register and residing in the catchment areas of: Stavanger and Fonna, 2) aged between 13 and 65 years, 3) meets diagnostic criteria for prodromal syndrome SIPS criteria [40], 4) does not meet current or life-time criteria for any psychotic disorder, 5) the symptoms are not better accounted for by an axis I, axis II or substance use disorder with the exception of schizotypal personality disorder (the presence of any of these disorders in itself is not an automatic reason for exclusion), 6) does not use any antipsychotic medication currently and have not used antipsychotic medication (regardless of dosage) for more than four weeks lifetime, 7) absence of known neurological or endocrine disorders that may have caused the presenting psychotic symptoms, 8) the patient is not mentally retarded with an IQ below 70, 9) is able to understand and speak Norwegian, 10) understood and signed an informed consent or assent for minors’ document.

The study was approved by the Regional Committee for Medical Research Ethics Health Region West (015.03). Written informed consent was obtained from all study participants. Parents or legal guardians gave written informed consent for patients younger than 18 years of age.

### Clinical assessments

As described in detail previously, clinical interviews were conducted by trained psychologists, psychiatric residents or psychiatrists—see study protocol for full description [41]. Diagnoses were reached using the Structured Clinical Interview for the DSM (SCID). Reliability of SCID in the TIPS study was satisfactory at kappa = .76 at baseline 1997–2000 [42], and at kappa = .9 in 2012 [43]. Regular reliability trainings are undertaken to avoid drift.

The matched (age and gender) healthy controls had no axis I or II psychiatric disorder (MINI interview), no known family history of psychiatric disorders in first-degree relatives, no current drug abuse or a past history of drug dependence other than nicotine consumption (MINI interview and AUDIT/DUDIT). As in previous studies [19, 44], healthy controls had higher scores on IQ-estimates than patients, as indicated by significantly higher scores on WAIS-III subtests (Digit-Symbol Coding and Vocabulary, but not on Letter-Number Sequencing, Digit Span or Block Design).

### Neuroimaging

MR imaging was performed with a 1.5 tesla scanner (450 Discovery; GE Medical Systems) equipped with a standard head-and-spine (HNS) coil.

#### Structural images

A structural image was acquired after the functional imaging using a BRAVO sequence (GE Healthcare); a T1-weight 3D IR-prepared spoiled gradient echo-sequence with the following parameters: TR/TE/TI = 7.9 ms / 3.1 ms / 450 ms, FOV = 24 x 19.2 cm^2^. Number of slices was 180 with a thickness of 1 mm. The in-plane sampling matrix was set to 240 x 192 resulting in an iso-tropic resolution of 1 mm^3^. The total scan time was 6:03 min.

#### Functional magnetic resonance imaging

Participants underwent fMRI scanning while performing a numerical n-back WM task as used in previous studies [23, 45, 46]. The task contains two conditions: (1) in the “2-back” (active) condition, participants were required to press a button when the number they saw matched the number seen two numbers before; and (2) in the “0-back” (baseline) condition, participants had to respond with a button press each time they saw the number zero. Numbers between 0 and 9 were displayed for 500 ms with an inter-trial interval of 900 ms. Each block consisted of 22 stimuli containing three targets and was indicated by an instruction cue displayed for 2 s before each block. Stimulation blocks and resting periods alternated within the experiment with a total of six 2-back and six 0-back blocks. During resting periods, participants were instructed to fixate on a cross in the center of the screen.

fMRI was performed using gradient-echo echo-planar imaging (TR, 2600 ms; TE, 35 ms; flip angle, 90°; matrix, 64 × 64; voxel size, 2 × 4 × 5 mm). The combination of a stimulus onset asynchrony of 1400 ms (500 ms stimulus duration; 900 ms intertrial interval) and a TR of 2600 ms resulted in 13 possible time points of stimulus presentation per TR. Across multiple stimulus presentations, this yielded an effective sampling rate of 5 Hz. Twenty-four slices approximately parallel to the bicommissural plane (anterior commissure–posterior commissure plane) were collected, covering the whole brain. Twenty fMRI volumes were acquired per block: 12 during stimulation (2-back or 0-back) and eight during the resting period. Blocks were presented alternately three times in each of the two runs (A B A B A B). A total of 240 volumes were collected.

Stimulus administration was controlled by E-Prime software (Psychology Software Tools Inc., Pittsburgh, PA) and the subjects’ response was given with their right index finger using standard response grips (Nordic Neuro Lab, Bergen, Norway).

### Data analysis

#### Image pre-processing

Functional MRI data was pre-processed and analysed with SPM12 (http://www.fil.ion.ucl.ac.uk/spm/) installed in the MATLAB R2012b environment (MATLAB version 8.0.0.783. Natick, Massachusetts: The MathWorks Inc., 2012). The first three volumes of each functional time series were discarded to remove non-steady-state effects caused by T1 saturation. Before pre-processing, the origin of the functional time series was set to the anterior commissure. Volumes were realigned to a mean image of both functional time series to correct for between-scan movements. The mean image was normalized to the standard EPI template provided by the Montreal Neurological Institute (MNI), and the parameters obtained in the normalization matrix were applied to the realigned images, which were resliced with a voxel size of 4 × 4 × 4 mm. Finally, all images were smoothed with a Gaussian kernel (full width at half-maximum, 8 mm).

#### Statistical parametric mapping

Statistical analysis was performed in the context of the general linear model (GLM) using a two-level approach [47]. On the first level of analysis, condition blocks of 2-back and 0-back were modelled. To account for movement-related variance, the realignment parameters were added as regressors in the design matrix. Contrast images for 0-back versus baseline and 2-back versus baseline were computed and taken to the second level for random-effects inference. On the second level, a mixed ANOVA with task (2-back/0-back) as within-subject factor and the between-subject factors group (healthy controls/CHR patients) was performed. The main effect of task is reported at a significance level of p < 0.05, FWE-corrected across the whole brain and a cluster extent of > 0 voxels. The interaction of interest (task by group) is reported at p < 0.05, FWE-corrected across the whole brain. All reported results remained significant when including age and gender as covariates. Based on the available research, PFC, PC, MTL and striatum were defined as a priori regions of interest (ROI). ROI analyses were carried out employing the WFU Pick Atlas toolbox [48, 49].

Structural MRI data was pre-processed employing standardized steps as implemented in the Freesurfer software (version 5.3, http://surfer.nmr.mgh.harvard.edu/) yielding normalized measures of cortical thickness for each subject [50–55]. The data was then smoothed with FWHM of 10 mm. To assess stability of the results, the analysis described below was repeated with FWHMs of 5 and 15, yielding consistent clusters.

A surface-based analysis estimating difference in cortical thickness between CHR and healthy controls was conducted employing generalized linear model as implemented in the software via the graphical user interface Qdec.

Multiple comparisons correction was carried out using Monte Carlo method. Cluster limits were generated with simulations as implemented in the Freesurfer software. Maps were tested against an empirical null distribution of maximum cluster size with 5,000 Gaussian noise simulations with a cluster-forming threshold of p < 0.005.

Volumetric analyses have been conducted for hippocampal volumes extracted via Freesurfer segmentation pipeline. Estimation of the between-group differences was accomplished step-wise in R programming language employing 1) two-sample T-tests and then 2) linear modelling using intracranial volume as a covariate.

## Results

### Demographic and clinical characteristics

The CHR sample (n = 41) had a mean age of 16.7 years and included 61% females (n = 25). Demographic and clinical characteristics of the sample are presented in Table 1. The healthy controls (n = 37) had a mean age of 16.9 years and included 51% females (n = 19). All patients and controls were of Nordic heritage except 2 CHR (European). When compared for alcohol and drug use on the AUDIT and DUDIT scales [56, 57] there were no significant differences (Chi-square test) for either alcohol or other substances, 94.3% of the CHR patients reported never to have used illegal substances versus 94.6% in the healthy controls group.

**Table 1:** Participant demographics and clinical measures.

	CHR (n = 41). Mean (SD)	Healthy controls (n = 37). Mean (SD)	Analysis
**Age**	16.7 (2.4)	16.9 (3.0)	t = -0.35, P = 0.73
**Gender—% male**	39%	49%	χ^2^ = 1.28, P = 0.26
Years of education	10.3 (1.8)	10.8 (2.5)	t = -1.00, P = 0.32
***DSM-IV diagnoses***	296.xx depressive: 11		
	296.89 Bipolar II: 1		
	309.xx Adjustment disorder: 6		
	300.xx Anxiety disorder: 8		
	300.3 OCD: 3		
	298.9 Psychotic disorder NOS: 2		
	299.8 Atypical autism: 1		
	300.4 Dysthymia: 1		
	304.8 Polysubstance dependence: 1		
	313.9 Disorder of infancy, childhood, or adolescence nos: 1		
	No diagnosis: 6		
**SIPS positive**	10.1 (3.3)		
**SIPS negative**	10.9 (6.3)		
**SIPS disorganized**	3.3 (2.4)		
**SIPS general**	8.8 (3.6)		
PANSS symptoms*			
**PANSS positive**		7.0 (0.2)	
**PANSS negative**		7.5 (0.6)	
**PANSS general**		16.0 (0.2)	
**PANSS total**		30.5 (0.7)	
***GAF-f***	49.9 (11.9)	90.4 (0.6)	t = -20.7, P < 0.01

### Behavioral performance

ANOVA with sensitivity index d’ revealed a significant main effect of task-load (F = 118.74; df = 1; p < 0.001). This indicated a reduction in performance during 2-back compared with the 0-back condition. There was no significant main effect of group (F = 0.259; df = 1; p = 0.61) and no significant task by group interaction (F = 0.238; df = 1; p = 0.63).

ANOVA with response times revealed a significant main effect of task (F = 157.78; df = 1; p < 0.001). This indicated slower reaction times during 2-back compared with the 0-back condition. There was no significant main effect of group (F = 0.163; df = 1; p = 0.69) and no significant task by group interaction (F = 0.28; df = 1; p = 0.87).

### Brain activity during WM

**Fig 1:**
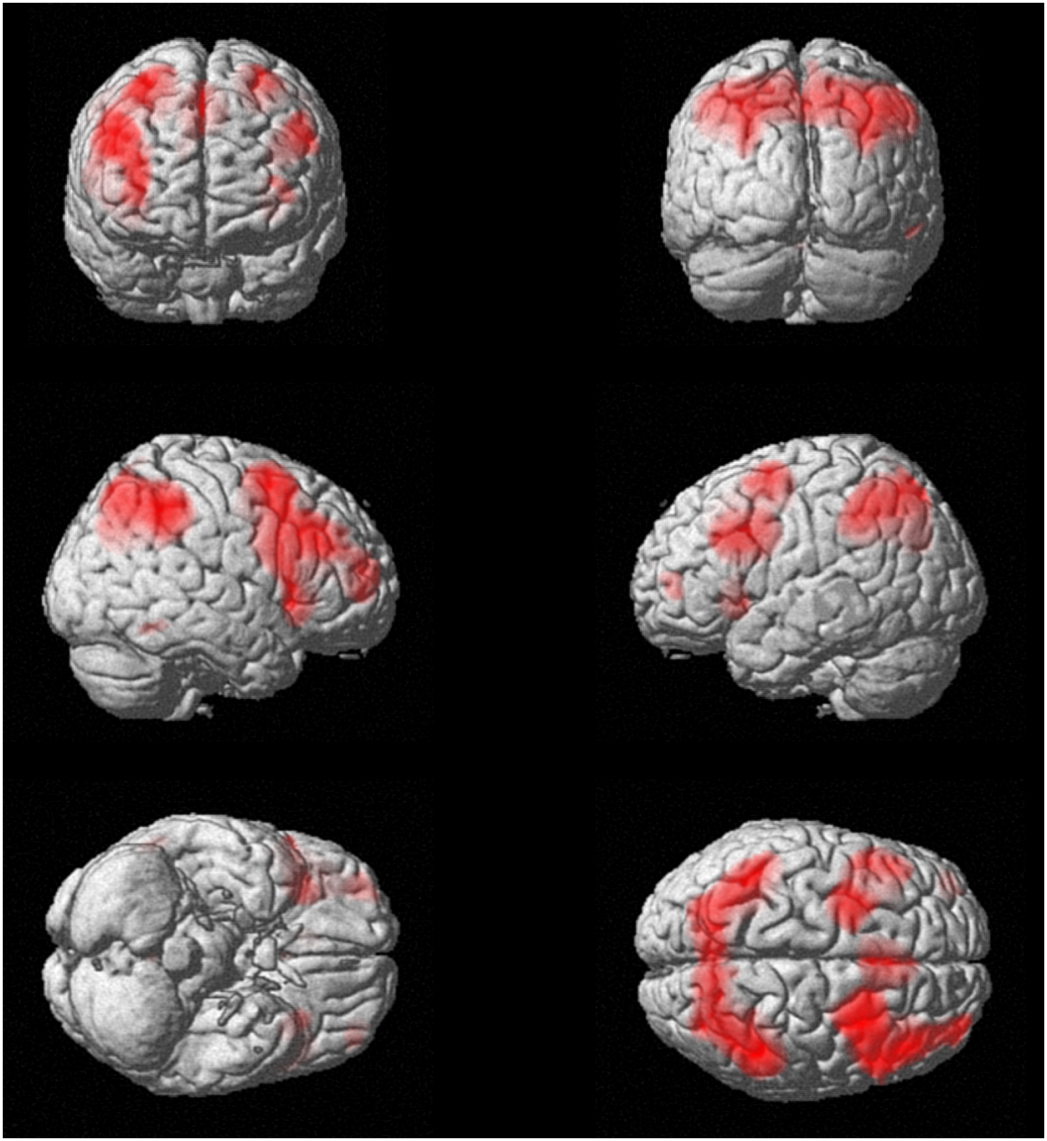
WM network. Regional brain activation during working memory across both groups as reflected by the contrast: (2back patients + 2back controls) > (0back patients + 0back controls).

There was a significant main effect (p < 0.05, whole brain FWE-corrected) of task in the WM-network including right superior frontal gyrus, bilateral middle frontal gyrus, inferior frontal gyrus, cingulate gyrus, hippocampus and parahippocampal gyrus, inferior parietal lobule, middle temporal gyrus, cuneus and cerebellum, in healthy controls and CHR patients. These regions included our main regions of interest, PC and dlPFC (Fig 1, Table 2).

**Table 2:** Regional brain activation during working memory.

Region	Brodmann area	*x*	*y*	*z*	Cluster size	*F* value
***Main effect of task***						
Superior frontal gyrus	BA9	12	56	38	10	27.97
Middle frontal gyrus	BA6	28	8	52	8047	143.32
Middle frontal gyrus	BA6	34	22	-2		130.42
Middle frontal gyrus	BA8	0	18	46		125.27
Middle frontal gyrus	BA9	-42	6	32	2154	103.88
Middle frontal gyrus	BA9	-48	24	32		90.77
Insula	BA13	-32	22	-4		86.58
Middle frontal gyrus	BA6	-28	0	58	1083	140.80
Middle frontal gyrus	BA10	-44	52	2	105	38.80
Inferior frontal gyrus	BA45/46	-54	30	10	9	27.28
Inferior frontal gyrus	BA47	32	36	-10	11	35.65
Cingulate gyrus	BA31	-4	-28	46		97.76
Precentral cortex	BA6	48	-14	56	9966	119.01
Premotor cortex	BA4	46	-20	62		113.34
Hippocampus	BA28	-24	-18	-18	8466	142.95
Inferior frontal gyrus	47	-26	34	-12		118.18
Parahippocampal gyrus	BA36	-26	-32	-16		101.01
Precentral cortex	BA3/4	-32	-26	70	1067	68.50
Precentral cortex	BA3/4	-20	-24	76		64.43
Postcentral cortex	BA3	-52	-18	54		55.04
Precentral cortex	BA4	-38	-18	40	31	29.35
Inferior parietal lobule	BA40	46	-42	46	8955	195.70
Inferior parietal lobule	BA40	-32	-52	40		184.85
Inferior parietal lobule	BA40	40	-46	42		178.48
Middle temporal gyrus	BA39	-54	-68	26	191	36.23
Angular gyrus	BA39	-48	-76	30		32.33
Middle temporal gyrus	BA37	56	-56	-18	40	34.05
Cuneus	BA18	-16	-100	10	2444	189.16
Cuneus	BA18	16	-98	10		152.03
Cuneus	BA18	-2	-86	-6		69.37
Cerebellum		0	-52	-24	7	29.54
Cerebellum		46	-80	4	1	23.36
***Group by task interaction***						
Hippocampus	BA58/53	32	-4	-22	1	23.40

Reported are regions that show a significant WM activation for CHR patients and controls taken together as indicated by the main effect of task (at p < 0.05 whole-brain FWE-corrected, cluster extent > 0 voxels) and regions that display a group by task interaction (at < 0.05, whole-brain FWE-corrected).

**Fig 2:**
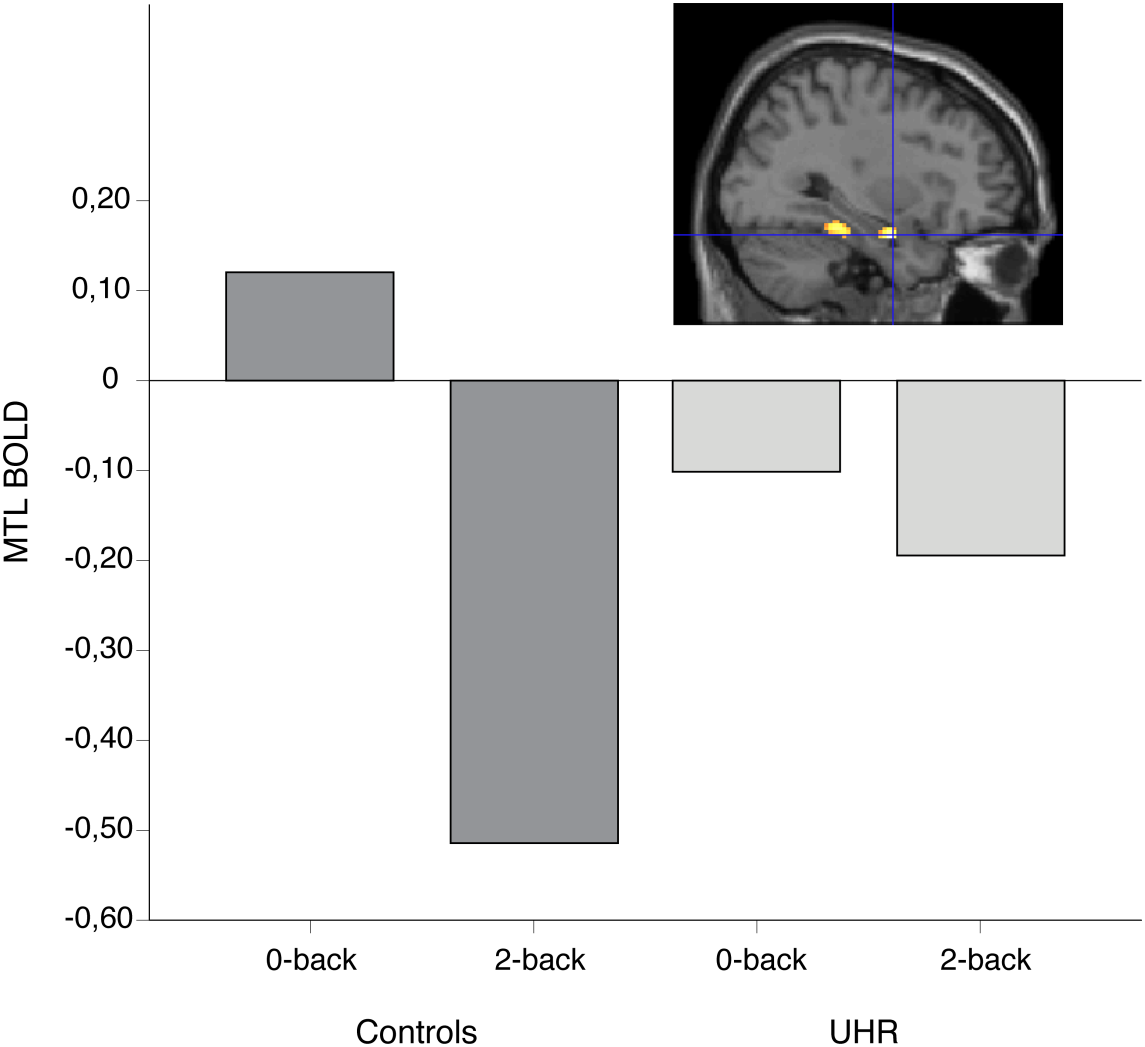
CHR participants showed significantly less WM-task induced suppression relative to controls in the right MTL (here displayed at p < 0.001 uncorrected). BOLD: blood oxygen level-dependent signal as measured by functional MRI (eigenvariate values extracted from voxel 32, -4, -22).

There was a significant (p < 0.05, whole brain FWE-corrected) task by group interaction in hippocampus in the right hemisphere (Fig 2). Using a less conservative approach (p < 0.001 uncorrected) the interaction contrast revealed that the peak activation in the right hippocampus (32, -4, -22) was part of an MTL cluster (k = 55) and caudally to this, yet another cluster (k = 83) in the right parahippocampal gyrus (peak voxel 34, -34, -18, F = 19.97). Post hoc t-tests revealed that suppression of the MTL was more pronounced in HC compared to CHR during 2-back vs. a matched baseline task. There was no significant activation in the task by group interaction in neither single prefrontal nor parietal ROIs.

### Cortical thickness

**Fig 3:**
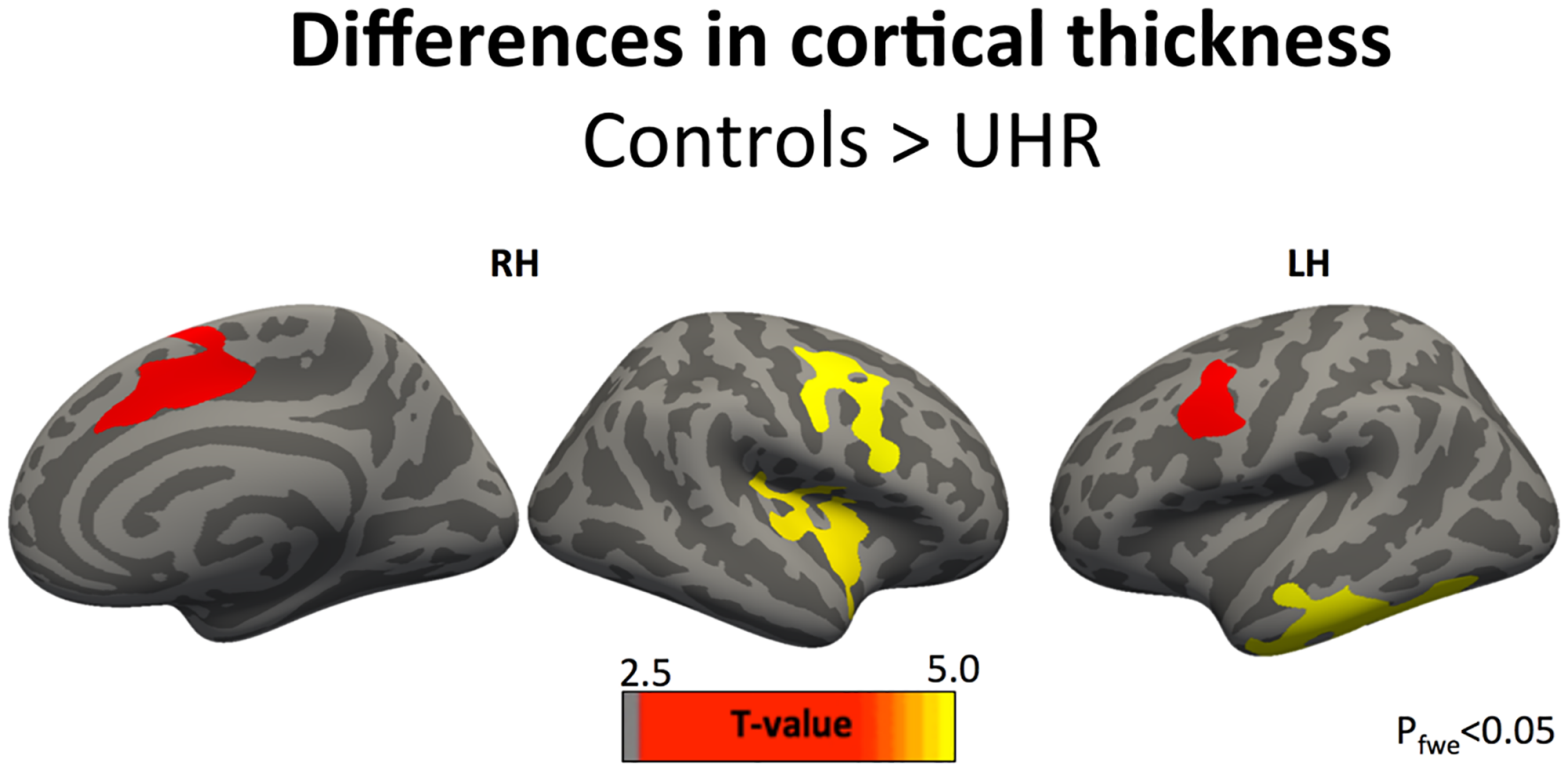
Surface-based analysis of cortical thickness: Contrast brain maps. The plot shows clusters of reduced cortical thickness in the CHR group compared to healthy controls. RH/LH—Left/Right Hemispheres.

We found that compared to healthy controls, CHR subjects demonstrated thinning in a number of cortical regions including dorsolateral prefrontal, dorsal cingulate, insular and inferior temporal areas with somewhat more pronounced differences observed in the right hemisphere (See Table 3, Fig 3)

**Table 3:** Surface-based analysis of cortical thickness.

HS	Size(mm^2)	TalX	TalY	TalZ	CWP	Cluster Annotation
LH	3225.33	-52.9	-28.3	-14.1	0.0001	Inferior temporal
LH	1086.14	-21.8	-46.5	-3.5	0.0034	DLPFC
RH	1499.53	36.4	-8.8	-7.7	0.0007	Insula
RH	1230.29	11	6	42.2	0.004	Dorsal cingulate
RH	1854.09	41.7	12.4	18.7	0.0001	DLPFC

HS—hemisphere (LH—left; RH—right).

Tal X,Y,Z—coordinates in the Talairach space.

CWP—cluster-wise p-value.

DLPFC—Dorsolateral Prefrontal Cortex.

#### Volumetric analysis of hippocampal volumes

**Fig 4:**
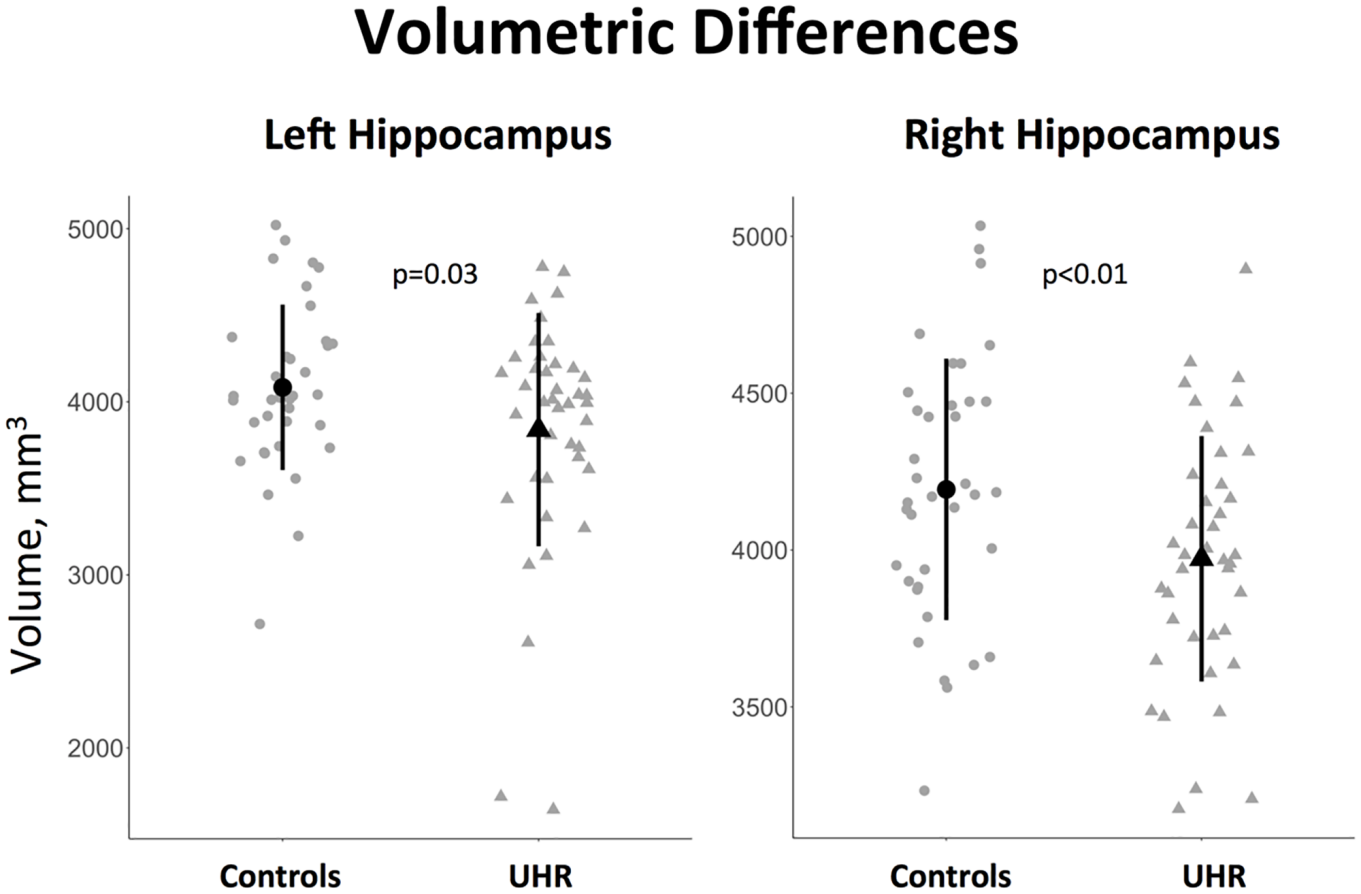
Volumetric differences. The figure shows between-group differences in hippocampal volumes. Individual values are plotted in grey; means (points) and standard deviations (bars) are plotted in black.

We found reduced hippocampal volumes in CHR subjects as compared to healthy controls with mean difference of 244.30 mm^3^ [T_one-tailed_ = 1.86 df = 72.135; p = 0.033] for the left and 221.19 mm^3^ [T_one-tailed_ = 2.41 df = 74; p<0.01] for the right sides (See Fig 4).

Adding intracranial volume into the model, however, diminished the observed effects of group (Left: T = 1.1,p = 0.28; Right: T = 1.37, p = 0.17).

As an exploratory step, we conducted a comparison of intracranial volume and found between-group differences [T_one-tailed_ = 2.08 df = 76; p = 0.02], which can be recognized as borderline-significant according to a strict Bonferroni-corrected p-value threshold (3 tests, p = 0.017).

## Discussion

In the present study, our aim was to examine potential differences in brain activity and/or structural abnormality in non-medicated CHR compared to healthy controls. We hypothesized that CHR patients would show altered brain activity in any of the areas corresponding to a fronto-temporo-parietal WM-network, but the results did not unequivocally support the hypothesis. The main finding with regard to functional MRI during an n-back task was that CHR was associated with altered functional activation of the MTL. Activity in the temporal lobe is not generally implicated in fMRI studies of working memory. Specifically, the medial temporal lobe has previously almost exclusively been associated with long-term memory function [58, 59]. More recent studies, however, indicate that the medial temporal lobe (MTL) may have a role in not only the encoding of information into long-term memory, but also in the maintenance of memory representations in a short-term or WM “buffer” [60] or as a primary hub of a default-mode network supporting memory processes [61]. The present findings are supported by a recently published study, indicating altered function of MTL in CHR compared to healthy controls [19]. A large multisite neuroimaging study of high risk individuals (n = 182) and matched controls (n = 167) showed that high risk individuals that later developed a psychosis had less gray matter volume in the parahippocampal gyrus than did the high risk individuals who did not later develop psychosis [62]. This reduction was seen contralateral to the same area (-21, 6, -27) as in the present study (32, -4, -22). Moreover, Valli et al. found that thalamic levels of glutamate are reduced in prodromal subjects and are negatively associated with activations in the prefrontal cortex, and positively with activation in the MTL regions during verbal fluency tasks [63]. Thus, both structural and functional as well as neurochemical imaging data lend support to the hypothesis that the MTL have a role in explaining a vulnerability to developing a psychosis.

In the study by Thermenos et al. [19] an increase of activity of the left parahippocampal in the CHR group as compared to healthy controls is reported as “hyperactivity” or, alternatively, “non-supression”. Interestingly the functional imaging results of the present study are in accordance with such an interpretation, displaying a significantly less pronounced WM-task induced suppression in the CHR group relative to controls in the right MTL. The present results, interpreted as an inability to suppress DMN regions during a task is often seen in psychiatric conditions [64, 65] and in CHR subjects in particular. For example, it has been shown in a study by Fryer et al. that while healthy controls showed WM load-dependent modulation of DMN suppression, CHR individuals had deficient higher-load DMN suppression that was similar to, but less pronounced than, the distributed suppression deficits evident in early schizophrenia patients [66]. These results suggest according to the authors that DMN dysregulation associated with schizophrenia predates psychosis onset.

Results from our surface-based analyses are in line with the reported functional findings, as well as with the previous studies consistently showing deficits within the prefrontal and temporal regions in CHR subjects [24, 25]. Similarly, we replicate previous reports showing reduced volumes of the hippocampus and its subfields in CHR subjects [67, 68]. It is worth mentioning, however, that these results were no longer significant after adjustment for intracranial volume, which itself appeared to differ between the studied groups. This observation supports a more generalized pathology associated with CHR that affects the brain in a substantially broader way than just targeting hippocampal formation. Taken together, the identified pattern of structural deficits may reflect perinatal and pubertal neurodevelopmental abnormalities leading to increased biological vulnerability to psychosis, such as impaired synaptic pruning and myelination [69, 70]. Meanwhile, despite meta-analytical evidences [3] not all studies report structural abnormalities in cross-sectional MRI analyses in CHR [26]. These inconsistencies may be driven by differences in the study populations as well as by the diversity of employed diagnostic criteria.

Our structural findings regarding reduced cortical thickness in bilateral DLPFC regions in CHR may seem contradictory to the lack of clear functional activation differences in these regions. DLPFC is a central component in working memory performance [71], correlated with cortical thickness in these areas. Thus, we would expect reduced performance as well as functional activation differences. A possible explanation for this is that symptom heterogeneity in the clinical sample might have been too large to reveal functional differences at a group level.

A limitation with the present study is the now established fact that CHR subjects which are recruited from a help-seeking populations, have increased risk due to sampling bias [72], which may have confounded the study. Further, the study does not provide novel hypotheses or findings, but rather validates and replicates earlier findings regarding CHR. The negative findings, with a lack of functional effects in prefrontal areas, should be interpreted with caution due to relatively low statistical power. Finally, our included subjects are relatively young (16.7 years on average) and may not be representative for a CHR population with the highest risk of conversion to psychosis, as the mean age for first episode psychosis is above 20 years of age in most samples.

To summarize, CHR was in the present study associated with robust structural brain changes concerning cortical thickness as compared with healthy controls and with a reduced suppression of MTL activation during a working-memory task, possibly reflecting a dysregulation of the default-mode network in the CHR group. Future studies should further investigate the role of abnormal MTL activation during working memory tasks as a marker for psychosis development.
